# Functional gradients of the medial parietal cortex in a healthy cohort with family history of sporadic Alzheimer’s disease

**DOI:** 10.1186/s13195-023-01228-3

**Published:** 2023-04-19

**Authors:** Dániel Veréb, Mite Mijalkov, Yu-Wei Chang, Anna Canal-Garcia, Emiliano Gomez-Ruis, Anne Maass, Sylvia Villeneuve, Giovanni Volpe, Joana B. Pereira

**Affiliations:** 1 Department of Neurobiology, Care Sciences and Society, Division of Clinical Geriatrics, Karolinska Institutet, Stockholm, Sweden; 2 Department of Physics, Goteborg University, Goteborg, Sweden; 3 German Center for Neurodegenerative Diseases (DZNE), 39120 Magdeburg, Germany; 4 Douglas Mental Health University Institute, McGill University, Montreal, QC Canada; 5 McConnell Brain Imaging Center, Montreal Neurological Institute, McGill University, Montreal, QC Canada; 6 Memory Research Unit, Department of Clinical Sciences Malmö, Lund University, Lund, Sweden

**Keywords:** Alzheimer’s disease, Cognitive aging, fMRI, Cerebrospinal fluid markers, Apolipoprotein E, Memory

## Abstract

**Background:**

The medial parietal cortex is an early site of pathological protein deposition in Alzheimer’s disease (AD). Previous studies have identified different subregions within this area; however, these subregions are often heterogeneous and disregard individual differences or subtle pathological alterations in the underlying functional architecture. To address this limitation, here we measured the continuous connectivity gradients of the medial parietal cortex and assessed their relationship with cerebrospinal fluid (CSF) biomarkers, ApoE ε4 carriership and memory in asymptomatic individuals at risk to develop AD.

**Methods:**

Two hundred sixty-three cognitively normal participants with a family history of sporadic AD who underwent resting-state and task-based functional MRI using encoding and retrieval tasks were included from the PREVENT-AD cohort. A novel method for characterizing spatially continuous patterns of functional connectivity was applied to estimate functional gradients in the medial parietal cortex during the resting-state and task-based conditions. This resulted in a set of nine parameters that described the appearance of the gradient across different spatial directions. We performed correlation analyses to assess whether these parameters were associated with CSF biomarkers of phosphorylated tau_181_ (p-tau), total tau (t-tau), and amyloid-ß_1-42_ (Aß). Then, we compared the spatial parameters between ApoE ε4 carriers and noncarriers, and evaluated the relationship between these parameters and memory.

**Results:**

Alterations involving the superior part of the medial parietal cortex, which was connected to regions of the default mode network, were associated with higher p-tau, t-tau levels as well as lower Aß/p-tau levels during the resting-state condition (*p* < 0.01). Similar alterations were found in ApoE ε4 carriers compared to non-carriers (*p* < 0.003). In contrast, lower immediate memory scores were associated with changes in the middle part of the medial parietal cortex, which was connected to inferior temporal and posterior parietal regions, during the encoding task (*p* = 0.001). No results were found when using conventional connectivity measures.

**Conclusions:**

Functional alterations in the medial parietal gradients are associated with CSF AD biomarkers, ApoE ε4 carriership, and lower memory in an asymptomatic cohort with a family history of sporadic AD, suggesting that functional gradients are sensitive to subtle changes associated with early AD stages.

**Supplementary Information:**

The online version contains supplementary material available at 10.1186/s13195-023-01228-3.

## Introduction

The medial parietal cortex is a structurally and functionally heterogeneous area that plays an integral role in brain function. In addition to being a hub of the default mode network, this area has been found to be active during a diverse range of cognitive processes, such as episodic memory [1], spontaneous thought [2], or language [3]. Due to its heterogeneity, many studies have identified different subregions within this area, including the precuneus, the posterior cingulate, and the retrosplenial cortex [4, 5]. However, these subdivisions do not capture the complexity of the medial parietal cortex because each of them is connected to different brain areas. Moreover, these subdivisions are usually obtained from an average group of healthy young participants, disregarding individual differences and possible pathological alterations. Recent evidence shows that functional connectivity changes across a spatially continuous manner rather than in discrete or separate brain areas [6]. Thus, an approach that maps these profiles as continuous connectivity gradients might be more sensitive in capturing functional reorganization and subtle early changes associated with neurodegenerative diseases.

Although several studies have assessed the functional gradients of different cortical and subcortical areas [7–9], the functional gradients of the medial parietal cortex remain unknown. The integrity of the medial parietal cortex is especially important for Alzheimer’s disease (AD), where this region is particularly vulnerable to the accumulation of pathological protein aggregates such as amyloid-ß and tau [10]. For instance, previous studies have found that amyloid accumulation starts in the precuneus and posterior cingulate in the early stages of AD [11], whereas tau pathology preferentially accumulates in the retrosplenial cortex due to its strong connections to the hippocampus and parahippocampal gyrus, which show early tau deposition [12]. Thus, it is possible that these pathological alterations result in early functional reorganization of the medial parietal cortex that could be captured using functional connectivity gradients.

In this study, we sought to determine whether the functional connectivity gradients of the medial parietal cortex are sensitive to markers associated with AD in a cohort of cognitively unimpaired participants with at least one parent or two siblings affected by this disorder [13]. In particular, these markers consisted of cerebrospinal fluid (CSF) amyloid-ß_1-42_ (Aß) levels, in addition to phosphorylated tau_181_ (p-tau_181_) and total tau (t-tau), apolipoprotein E (ApoE) ε4 carriership, and memory. Reductions in CSF concentrations of Aß have been shown to occur 25 years before the onset of dementia in familial AD, being followed by increases in soluble tau measured by p-tau, and later by t-tau, which is thought to reflect neurodegeneration [14]. The ratio of CSF Aß/p-tau has also been investigated as a marker of underlying amyloid deposition and correlates well with early functional changes during the course of AD, such as default mode network dysfunction [15, 16]. The ApoE ε4 allele is the strongest genetic risk factor for developing sporadic AD, showing an association with functional connectivity since early disease stages [17] and lowering the age of disease onset [18]. Finally, memory decline is one of the first clinical symptoms of AD, which deteriorates slowly over time before reaching the threshold for overt impairment [19].

We hypothesized that alterations in the functional gradients of the medial parietal cortex would be more strongly associated with CSF AD biomarkers, ApoE ε4 allele carriership and memory compared to commonly used connectivity measures, providing support to the use of this novel imaging marker to detect early changes associated with the preclinical stage of AD.

## Methods

### Participants

Since no studies had previously assessed the functional gradients in the medial parietal cortex, first we identified these gradients in a cohort of healthy young individuals with high-resolution functional neuroimaging data from the Human Connectome Project (http://db.humanconnectome.org) [20]. In particular, we used the resting-state functional MRI data from the 100 Unrelated Subjects dataset, which consists of 46 men and 54 women between 22 and 36 years of age without any previous medical conditions or cognitive impairment. Then, we replicated these functional gradients on the functional MRI data from the Presymptomatic Evaluation of Experimental or Novel Treatments for Alzheimer’s Disease (PREVENT-AD) registered dataset [13], an observational cohort of cognitively unimpaired older adults with parental or multiple sibling relatives diagnosed with AD [21]. We used both resting state and task-based fMRI in this analysis since these modalities might provide complementary information about brain function [22]. The participants’ cognitive status was assessed using the Repeatable Battery for the Assessment of Neuropsychological Status (RBANS), a test assessing immediate and delayed memory as well as language, visuospatial functions, and attention. Participants with available structural and fMRI data as well as RBANS scores (*n* = 340) were included in the study. At the time of the measurements, all participants were cognitively normal.

### Image acquisition

Resting-state fMRI scans from the HCP cohort were acquired using a Siemens Skyra 3 T MRI scanner with a 32-channel head coil using a GE-EPI sequence with the following parameters: repetition time = 720 ms; echo time = 33 ms; field of view = 208 mm × 180 mm; matrix = 104 × 90; voxel size = 2 × 2 × 2 mm^3^; 72 slices; 1200 volumes. Participants underwent two separate sessions with scans of left-to-right and right-to-left phase encoding directions, resulting in four 4D volumes per participant.

Functional scans from the PREVENT-AD cohort were acquired on a Siemens TIM Trio 3 T MRI scanner at the Brain Imaging Centre of the Douglas Mental Health University Institute with a 12-channel head coil. Acquisition parameter details and imaging protocols are described in [13]. In short, resting-state and task-based fMRI scans were acquired with identical parameters, using a GE-EPI T2^*^-weighted sequence (repetition time = 2000 ms; echo time = 30 ms; field of view = 256 × 256 mm; voxel size = 4 × 4 × 4 mm; 32 slices; 150 volumes for the resting state scan, 185 volumes for the memory encoding task and 455 volumes for the memory retrieval task). For each participant, two runs of resting-state scans were acquired. During the encoding task, participants were asked to remember objects and their spatial location (left versus right) on a screen, whereas, during the retrieval task, they had to recognize whether the current stimulus was among those presented during the encoding task and whether its spatial location was congruent with the previous occurrence [23].

### Image preprocessing

The 100 Unrelated Subjects HCP functional images were preprocessed according to a minimal preprocessing pipeline [24]. First, functional MRI scans underwent EPI distortion correction, removal of non-brain tissue, motion correction, intensity normalization, and a two-stage registration to standard MNI space, followed by regression of noise and motion-related artifacts using FSL FIX (https://fsl.fmrib.ox.ac.uk/fsl/fslwiki/FIX) and high-pass filtering with a cut-off of 2000s. Additionally, following previous publications using functional gradients [7, 25], signals from the CSF and white matter were removed via nuisance regression, and the resulting volumes underwent spatial smoothing with an isotropic 3D Gaussian kernel of 6 mm full-width-at-half-maximum. Next, for each participant, the four available 4D resting-state volumes were individually mean-centered, normalized to unit standard deviation, and concatenated before further processing.

Functional MRI scans from the PREVENT-AD dataset were preprocessed via a standard pipeline implemented in fMRIPrep (v20.2.4). Resting-state and task-based scans were preprocessed using the same parameters to ensure the comparability of results. After removing the first two volumes to allow for steady-state magnetization, functional images were corrected for motion and slice timing effects, skull-stripped and co-registered to a standard 2 mm resolution MNI152 template space using a two-stage registration approach with Freesurfer [26] and ANTs [27]. Nuisance regression was performed to further correct for motion effects (employing the 24-parameter head motion model [28]) and to remove confounding signals from the CSF and white matter. Resulting volumes were subjected to high-pass filtering with a 0.01-Hz cutoff and spatial smoothing with a Gaussian kernel of 6 mm full-width-at-half-maximum. The two resting state scans were concatenated after mean-centering and individual normalization to unit standard deviation. Here we did not perform activation analyses of the task-based data; for further information about baseline task-related fMRI results, see [29].

### Defining the medial parietal cortex

**Fig. 1:**
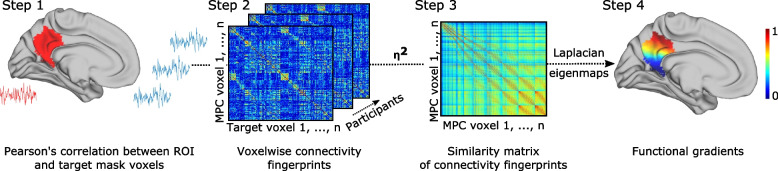
A schematic representation of the connectopic mapping procedure. After the delineation of the region of interest and target mask, functional connectivity was computed between the ROI and target mask voxels (Step 1), which resulted in a functional connectivity matrix, i.e., connectivity fingerprint for each ROI voxel (Step 2). These fingerprints are then compared, and a similarity matrix is built using the eta-squared distance measure (Step 3). Finally, the Laplacian eigenmap algorithm is used to decompose the graph-transformed similarity matrix, obtaining functional gradients (Step 4). Abbreviations: MPC, medial parietal cortex

To identify the bilateral posterior medial parietal cortex, we followed a previously described scheme [4]. Specifically, the postero- and antero-superior borders of this region of interest (ROI) were defined based on cytoarchitectonic areal boundaries using the probabilistic histological maps from the Jülich brain atlas [30], whereas the splenium of the corpus callosum and the parieto-occipital sulcus served as posterior and rostral borders. The final ROI therefore included the precuneus, posterior cingulate cortex, and retrosplenial cortex, as depicted in Fig. 1A.

### Estimation of functional connectivity gradients in the medial parietal cortex

We used connectopic mapping, a data-driven approach for characterizing spatially continuous changes of connectivity profiles in our medial parietal cortex ROI, to derive the direction or gradients of connectivity for all participants using their whole-brain connectivity profiles (see Fig. 1 for a flowchart of the analysis). The analysis was performed using the ConGrads toolbox (https://github.com/koenhaak/congrads). First, a “connectivity fingerprint” was acquired for each voxel in the ROI, which contains the Pearson’s correlation coefficients between the ROI voxel time series and voxels in the target mask (in this case, the whole brain). For computational efficiency, the voxel time series under the target mask underwent singular value decomposition (SVD), and the connectivity fingerprints were calculated using the SVD component time series [25]. Then, a similarity matrix was calculated between the fingerprints of each ROI voxel using the eta-squared measure [31]. Finally, the similarity matrix was transformed into a connected graph and decomposed using the Laplacian eigenmaps algorithm [32], yielding eigenvectors of the graph Laplacian that represent spatially continuous patterns of functional organization, i.e. the gradients. Voxels with similar connectivity profiles have similar gradient values.

To summarize the spatial layout of the gradients and make them eligible for statistical inference, we employed a spatial regression approach (trend surface modeling) that describes a functional gradient in terms of polynomial basis functions [33]. Fitting these functions produces several coefficients that describe the spatial features of the gradient that can be used for statistical analyses instead of doing voxel-wise analyses, which might reduce sensitivity. Here we used Bayesian linear regression to fit a third order trend surface model (TSM) that describes spatial properties of gradients along the three axes of MNI space in nine parameters (coefficients corresponding to basis functions describing *x*, *y*, *z* in addition to their second and third power: *x*^2^, *y*^2^, *z*^2^, and *x*^3^, *y*^3^, *z*^3^). In practical terms, first order parameters of the TSM characterize the slope of connectivity changes along the *x*-, *y*-, *z*-axes, whereas higher-order parameters describe more nuanced spatial features (e.g., curvature). As an example, constant stepwise connectivity changes along the gradient would result in increases in first-order and decreases in higher-order parameters, whereas a more clustered or complex functional organization would lead to diminished first-order and increased higher-order parameters. We chose the third model order based on previous studies and as a trade-off between model parsimony and explained variance, since the increase in explained variance using higher model orders was minimal [7, 34, 35]. In order to include biologically plausible gradients in the statistical analysis of the PREVENT-AD cohort, only participants whose dominant gradient preserved the main direction of the average HCP gradient were included for further analysis. Additionally, participants were only included if the trend surface model described at least 70% of the total variance in the spatial layout of the dominant gradient, similarly to previous studies [35]. This threshold was chosen based on the trend surface model and its ability to recapitulate the spatial features of the main gradient. When the model was not able to capture at least 70% variance, the main gradient was in most cases not biologically plausible, e.g., the specific eigenmap was driven by a small number of outlier voxels, resulting in a large uniform cluster occupying one extreme of the gradient scale and outlier voxels occupying the remainder of the scale, suggesting an artifact rather than disease-related gradient alterations (examples of such artifactual gradients are shown in Fig. S1 in the Supplementary material). This criterion was fulfilled in 263 out of 340 participants in the main PREVENT-AD cohort with resting-state fMRI data (77% of the participants). The excluded subjects did not differ from the analyzed sample in demographical variables (age: Student’s *t*-test, *p* < 0.39; sex: Fisher’s exact test: *p* < 0.092, education years: Student’s *t*-test, *p* < 0.89), ApoE ε4 frequency (Fisher’s exact test: *p* < 0.51), CSF marker levels (Student’s *t*-test, tau: *p* < 0.86; p-tau: *p* < 0.73; Aß: *p* < 0.25), or memory (Student’s *t*-test, age-adjusted immediate memory: *p* < 0.32; age-adjusted delayed memory: *p* < 0.36).

### Cerebrospinal fluid markers in the PREVENT-AD cohort

CSF samples were collected in a subsample of the PREVENT-AD cohort via lumbar puncture. Biomarker levels of p-tau, t-tau, and Aß were determined using an enzyme-linked immunosorbent assay with a European BIOMARK-APD validated standard protocol [13]. Demographic characteristics of the CSF subsample are described in Table 1.

**Table 1:** Characteristics of the PREVENT-AD cohort

	**Resting-state fMRI** **(*n* = 263)**	**Task-based fMRI** **(*n* = 149)**	**CSF** **(*n* = 93)**
Females/males	183/80	103/46	60/33
Age	63.25 (4.97)	63.43 (± 4.96)	62.52 (5.20)
Education years	15.49 (3.45)	15.32 (± 3.38)	15.28 (3.04)
ApoE ε4 carriers/noncarriers	99/164	52/97	34/59
RBANS immediate memory score	103.06 (11.07)	102.77 (11.03)	102.59 (10.67)
RBANS delayed memory score	101.69 (9.22)	101.15 (9.25)	100.02 (9.91)
CSF t-tau	-	-	279.99 (± 132.21)
CSF p-tau	-	-	48.39 (± 18.12)
CSF amyloid	-	-	1193 (± 243.65)
CSF amyloid/p-tau	-	-	28.04 (± 11.44)

Values represent means (standard deviations). *fMRI* Functional magnetic resonance imaging, *CSF* Cerebrospinal fluid, *ApoE* Apolipoprotein E, *RBANS* Repeatable Battery for the Assessment of Neuropsychological Status, *t-tau* Total tau, *p-tau* Phosphorylated tau, *amyloid* amyloid ß-1–42

### Genotyping in the PREVENT-AD cohort

ApoE ε4 carrier status was determined using pyrosequencing at the rs429358 and rs7412 loci after isolating DNA from 200 μl blood samples with a QIASymphony apparatus and the DNA Blood Mini QIA Kit [13]. Demographic characteristics of the ApoE ε4 carrier and non-carrier subsamples are described in Table 1.

### Statistical analysis

Statistical analyses were conducted in MATLAB (version R2021b, MathWorks, inc.). Due to the non-normality of the variables, the associations between CSF AD biomarkers and cognitive variables with the gradients spatial parameters were assessed using separate partial Spearman’s rank correlations, while controlling for age, sex in addition to years of education (for analyses using cognition). From the RBANS battery, we focused specifically on the age-adjusted immediate memory and delayed memory sub-scores, since these are the earliest affected domains in AD [19]. However, we also ran specificity analyses using the other RBANS cognitive domain scores. Differences in gradient parameters between Apoe ε4 carriers and noncarriers were assessed with Mann–Whitney *U*-tests, while controlling for age, sex, and education.

In order to assess the added value of the functional gradients on top of other common functional connectivity measures we performed secondary analyses to extract the intrinsic connectivity within the default-mode network, whole brain connectivity of the medial parietal cortex, as well as whole brain connectivity in three medial parietal subregions (precuneus, posterior cingulate cortex, and retrosplenial cortex) and investigated their relationship with CSF biomarkers, ApoE status and RBANS memory scores.

For all analyses, adjustment for multiple comparisons was performed by applying false discovery rate (FDR) corrections.

## Results

### Characteristics of the PREVENT-AD sample

The characteristics of the cohort can be found in Table 1. A total of 263 participants with resting-state functional MRI scans and 149 participants with encoding and retrieval task-based fMRI were included from the PREVENT-AD cohort. ApoE ε4 carrier status was determined as having at least one copy of the ε4 allele, resulting in 99 carriers and 164 noncarriers. Carriers and noncarriers did not significantly differ in age, years of education, RBANS memory subtest scores, or sex. A subset of 93 participants also had CSF levels of p-tau, t-tau, and Aß, which were analyzed in relation to the functional gradient features.

### The functional gradients of the medial parietal cortex in the HCP are reproducible in the PREVENT-AD cohort

**Fig. 2:**
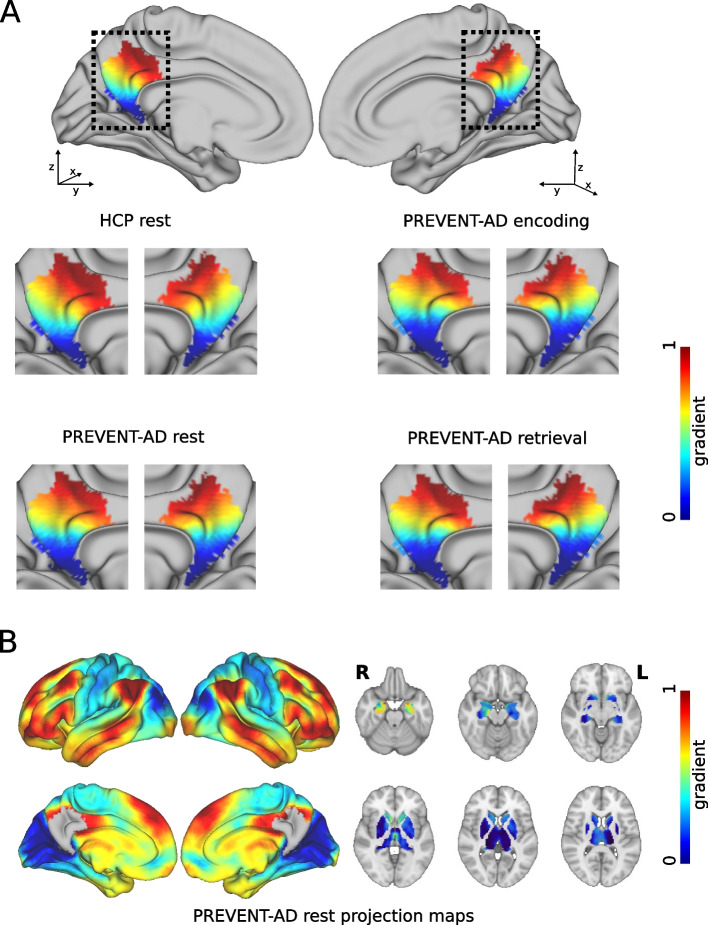
Medial parietal functional gradients in the HCP and PREVENT-AD cohorts. The average dominant gradients from the HCP and PREVENT-AD cohorts were projected onto the HCP 32 k vertex resolution surface template (first row). We expanded the medial parietal region in sagittal view to highlight the similarity in the functional gradients for the HCP cohort resting fMRI condition, PREVENT-AD cohort resting fMRI condition, PREVENT-AD cohort fMRI encoding task, and PREVENT-AD cohort fMRI retrieval task (second and third rows) (**A**). The spatial maps depicting the strongest connections from the medial parietal cortex to other cortical and subcortical areas in the PREVENT-AD cohort are shown below (**B**). In all figures, the color bars represent normalized gradient values

The dominant functional gradient consisted of a continuous change in connectivity profiles along the inferior to the superior brain axis in the HCP cohort. We observed very similar patterns at the group and individual levels in the PREVENT-AD cohort (Fig. 2), indicating these findings were robust. The connectivity profiles along this gradient ranged from connectivity to visual, thalamic, and posterior hippocampal areas in the inferior part of the medial parietal cortex (denoted by blue-light blue colors in Fig. 2A and B) to medial frontoparietal projections associated with the default mode network in the superior part of the medial parietal cortex (denoted by yellow–red colors in Fig. 2A and B). Since the dominant gradient revealed an inferior-to-superior axis, spatial parameters corresponding to this axis were the most prominent, indicating the largest stepwise changes in connectivity profiles along this direction (*z*-axis in MNI space). Importantly, stepwise changes were largely constant along the main gradient, as denoted by higher values of the first-order *z*-parameter. This suggests that functional connectivity changes in a continuous manner throughout the medial parietal cortex rather than being organized into separate and discrete subregions.

### Cerebrospinal fluid AD markers are associated with spatial gradient parameters

**Fig. 3:**
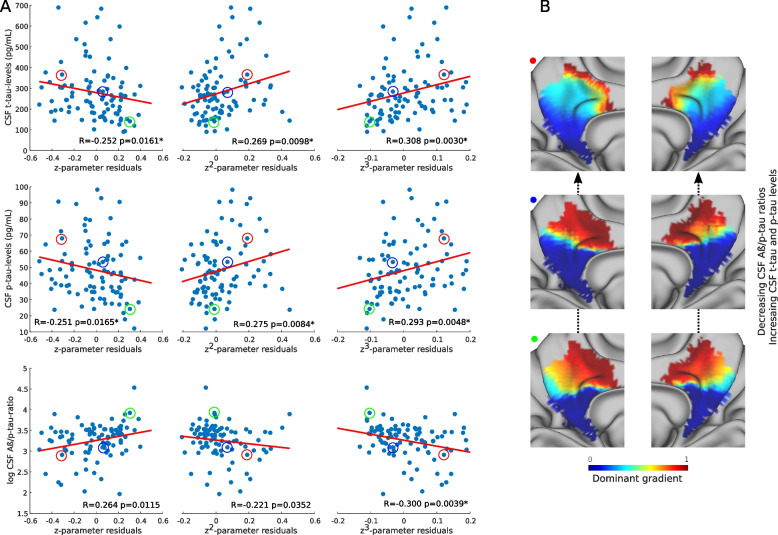
Resting-state gradient parameters correlate with cerebrospinal fluid AD biomarkers. Scatter plots showing correlations between phosphorylated tau (p-tau), total tau (t-tau), and amyloid-ß42/phosphorylated tau (Aß/p-tau) cerebrospinal fluid (CSF) levels with gradient parameters along the inferior-superior axis. Aß/p-tau ratios have been log-transformed for visualization. Correlations that remained significant after correction for multiple comparisons are marked with an asterisk (**A**). Individual participants from panel **A** are circled with different colors to show examples of what their functional gradients look like (**B**)

The spatial parameters of the medial parietal gradients during the resting-state condition were associated with CSF p-tau (*z*-parameter: *R* =  − 0.25, *p* = 0.017; *z*^2^-parameter: *R* = 0.28, *p* = 0.008; *z*^3^-parameter: *R* = 0.29, *p* = 0.005) and t-tau (*z*-parameter: *R* =  − 0.25, *p* = 0.016; *z*^2^-parameter: *R* = 0.27, *p* = 0.01; *z*^3^-parameter: *R* = 0.31, *p* = 0.003) levels (Fig. 3A). Similar associations were found between Aß and Aß/p-tau levels with the gradients during resting-state, with Aß showing a trend-level association (*y*^3^-parameter: *R* =  − 0.21, *p* = 0.047) (result not shown), whereas Aß/p-tau showed significant (*z*^3^-parameter: *R* =  − 0.30, *p* = 0.004) or trend-level associations with other z-axis parameters (*z*-parameter: *R* = 0.26, *p* = 0.012; *z*^2^-parameter: *R* =  − 0.22, *p* = 0.035) during resting-state (Fig. 3A). These associations with CSF biomarkers can be visualized in the three examples of individual gradient maps of participants that had both low, medium, and high p-tau and t-tau levels as well as high, medium, and low Aß/p-tau levels in Fi. 3B, indicating that a higher representation in the inferior blue part of the gradient and a smaller representation of the superior red portion are associated with higher tau and p-tau as well as lower Aß/p-tau levels. This reflects a reduction in first-order parameters and increases in higher-order TSM parameters along the z-axis that were accompanied by higher t-tau/p-tau and lower Aß/p-tau levels, indicating an imbalance of stepwise changes along the gradient that results in a more clustered functional organization. These findings are relevant in the context of AD since the superior part is connected to the main hubs of the default mode network.

The task-based gradients were not associated with any CSF biomarkers.

### ApoE ε4 allele carriers show differences in spatial gradient parameters compared to non-carriers

**Fig. 4:**
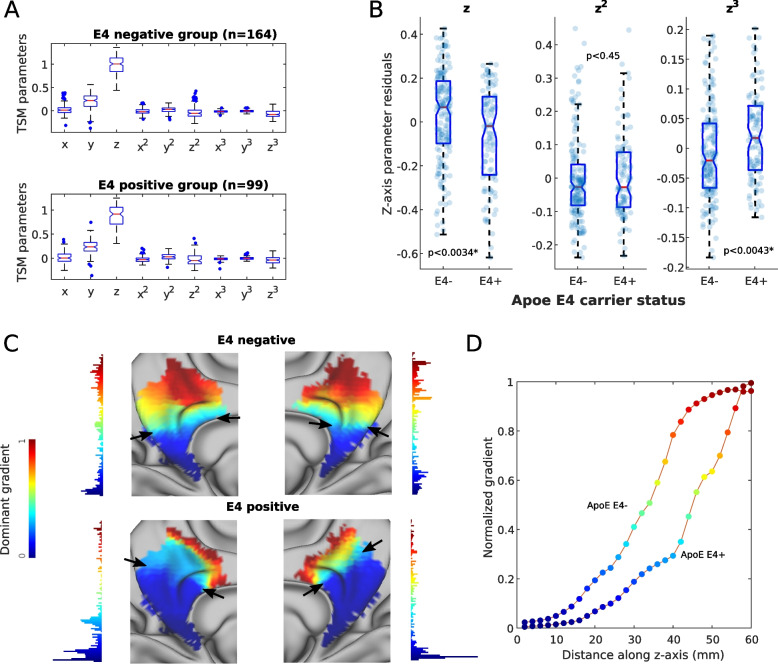
Differences in the main resting-state medial parietal gradient parameters between ApoE ε4 allele carriers and noncarriers. Boxplots showing the different trend surface model parameters (describing spatial variation in the gradients) in the ApoE ε4 carrier and noncarrier groups (**A**) and parameters showing significant differences after correcting for the effects of age, sex, education, and multiple comparisons between groups (**B**). Surface projections of the dominant gradient in a representative ApoE ε4 noncarrier and a ApoE ε4 carrier participant with black arrows highlighting where the main differences lie between the two participants (**B**). The color bars besides the projections denote normalized gradient values, whereas the histograms depict the distribution of voxels along the gradient scale (**C**). Plots depicting gradient values along the inferior-superior axis after projection to a streamline of the gradient field for a ApoE ε4 noncarrier and a ApoE ε4 carrier participant (**D**). Abbreviations: TSM, trend surface model; ε4 − , ApoE ε4 noncarrier; ε4 + , ApoE ε4 carrier

The distribution of spatial model parameters summarizing the layout of the dominant gradient in the ApoE ε4 allele carriers and noncarrier groups is depicted in Fig. 4A. There was a significant difference between the main resting-state gradients of ApoE ε4 allele carriers and noncarriers (*z*-parameter: *p* = 0.003; *z*^3^-parameter: *p* = 0.004, see Fig. 4B) which again reflected an increased representation of the inferior part as well as reduced representation of the superior part of the medial parietal cortex gradients in the ApoE ε4 carriers (Fig. 4C). For an example of gradient values along the main gradient streamline in representative participants in the two groups, see Fig. 4D. The task-based gradients did not show significant differences between the two groups.

### Memory is associated with different spatial gradient layouts

**Fig. 5:**
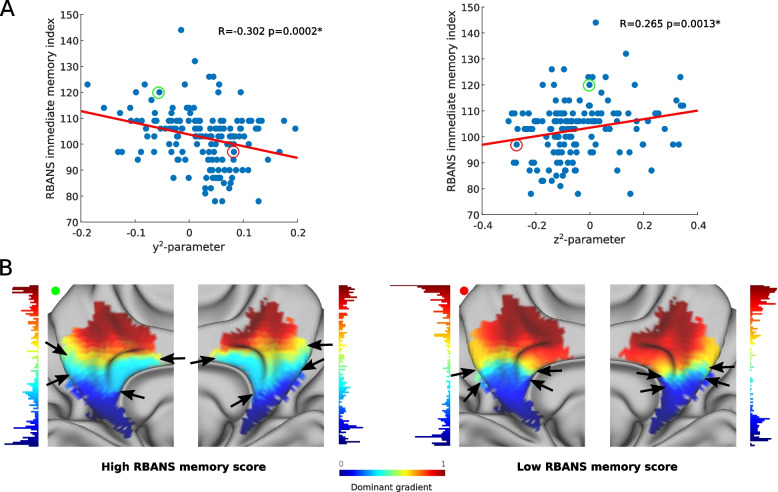
Immediate memory is associated with the gradient functional parameters from the fMRI task-based encoding task. Scatter plots showing significant associations between RBANS immediate memory scores and relevant trend surface model parameters (**A**). Two individual participants with high and low memory scores are circled in different colors in the scatter plots (**A**) and their representative functional gradients are shown below (**B**) with black arrows highlighting where the main differences lie between the two participants (namely, participants with better memory performance exhibited a more evenly distributed gradient with similar stepwise changes in functional profiles across the medial parietal cortex — similarly to resting state gradients in participants with lower CSF marker levels — whereas the gradient was steeper in the middle portion for participants with lower memory scores, essentially partitioning the medial parietal cortex into two functionally distinct clusters). The histograms next to the gradients depict the distribution of voxels along the gradient scale

The resting-state and retrieval task-based gradients were not associated with cognition. In contrast, spatial features of the encoding fMRI task were associated with the immediate memory RBANS sub-scores (Fig. 5A). As illustrated in Fig. 5B, which shows the functional gradients of two individual participants with high and low memory scores, gradient differences were particularly prominent in the middle part of the medial parietal cortex (depicted in light blue), which is mainly connected to inferior temporal areas and posterior lateral parietal regions and was reduced in individuals with low memory scores (*y*^2^-parameter: *R* =  − 0.31, *p* = 0.001; *z*^2^-parameter: R = 0.265, *p* = 0.001). No associations were found with the other cognitive domains.

### Functional gradients are more sensitive to early AD changes compared to common connectivity measures

In addition to the continuous functional gradients of the medial parietal cortex, we calculated intrinsic connectivity within the default mode network, whole brain connectivity, and connectivity in the three subregions; precuneus, posterior cingulate, and retrosplenial cortex. None of these analyses revealed associations with CSF markers, ApoE status, or RBANS memory scores (for full details and results see Table S1 in the Supplementary Material), indicating that the functional gradients provide additional information that cannot be captured by conventional connectivity measures.

## Discussion

In this study, we assessed the continuous functional gradients of the medial parietal cortex based on their connectivity with other cortical and subcortical areas in an asymptomatic cohort at risk for developing sporadic AD. In contrast to commonly used connectivity measures, we found that the continuous functional gradients were closely associated with CSF biomarkers, ApoE ε4 and memory, which play an important role in AD. These gradients were organized along the inferior to superior axis of the medial parietal cortex and were reproducible across cohorts. These findings indicate that the functional medial parietal gradients are a robust measure that can be used to detect subtle changes associated with AD and could potentially be used as an alternative imaging marker for very early disease stages since the observed gradient changes reflect a biomarker profile that is associated with an increased risk of developing AD (higher p-tau/tau and lower Aß).

The organization we found in the continuous functional gradients represents a natural spatial shift in connectivity profiles of the medial parietal cortex. This organization shows that the gradients are more distinct and heterogeneous along the inferior to superior axis because they are connected to different brain areas along this axis. Since more diverse functional gradients represent a healthy characteristic of brain connectivity, we can assume that a loss of this diversity represents a pathological change associated with damaged connectivity between the medial parietal cortex and other brain areas, in line with previous research showing that higher Aß and tau levels bring about a dedifferentiation (loss of within-network and increase of between network connectivity, as well as decreased segregation) in functional memory networks [36, 37]. Specifically, higher CSF levels of p-tau and t-tau were associated with a greater representation of the inferior part of the medial parietal gradient, which expanded to include the posterior cingulate in addition to the retrosplenial cortex. Both of these regions are affected during the course of AD [38]. Consequently, the superior part of the gradient that connects the medial parietal cortex to the main hub regions of the default-mode network was less extended. So, altogether, the alterations we found in the medial parietal gradients are in line with previous studies reporting functional changes in the precuneus in the course of AD; however, to our knowledge, our study is the first to show alterations in medial parietal functional gradients in the preclinical stage. Although it has been reported that elderly individuals exhibit higher levels of t-tau as a function of age [39], in our study the correlations with t-tau and p-tau remained significant when controlling for age, suggesting that the associations with AD-related tau/p-tau changes were at least partially independent of age. The loss of functional diversity we found in the medial parietal region might be in part due to reduced connectivity between the retrosplenial cortex and the hippocampus known to occur early during the disease course and in association with increased CSF p-tau levels [40]. Indeed, the retrosplenial cortex has specific connections to the hippocampus and is considered a gateway between subcortical and cortical subsystems of the default mode network that relays information to the posterior cingulate cortex [41]. The pathological involvement of specific subcortical connections might disrupt the normal flow of information that results in a less structured functional organization and a loss of specialization. In animal models, tau deposition was associated with increased neuronal excitability and excitotoxicity [42], possibly through a combination of increased oxidative stress and cytoskeletal alterations, resulting in mitochondrial dysfunction and amplified calcium-related signaling cascades [43]. Increased excitability and excitotoxicity in turn can cause disturbances in neuronal synchronization that leads to reduced connectivity [44]. As such, the loss of diversity in the medial parietal gradients we observed in the current study might be a downstream effect analogous to the cascading network failure model of AD, which postulates that the posterior DMN is among the first affected networks that demonstrate connectivity loss even in the absence of detectable amyloid deposition [45]. Accordingly, we found only subtle associations between CSF Aß levels and resting-state gradient features. However, we observed a significant link between lower CSF Aß/p-tau ratio and loss of diversity reflecting reduced connectivity between the superior part of the medial parietal cortex with other regions of the default-mode network. The CSF Aß/p-tau ratio has been shown to provide better diagnostic accuracy for distinguishing AD patients from healthy controls and other forms of dementia than the two separate measures [46]. Furthermore, it has been shown to correspond well to brain amyloid deposition [16] and is associated with decreased connectivity within the default mode network in AD [15]. It has also been suggested that CSF Aß/p-tau ratio predicts future conversion to dementia in cognitively normal older adults [47]. Gradients associated with worse CSF biomarker profiles also exhibited changes in the superior part of the medial parietal cortex, which might be due to an overall loss of connectivity in the default mode network. Since these areas are mainly connected to the parietal and prefrontal cortices, it is possible that the early disconnection between the posterior and anterior subsystems of the default-mode network manifests in the observed gradient changes [45]. Together, these results suggest that the normal functional organization of the medial parietal cortex is already undergoing subtle changes in individuals at risk for developing sporadic AD that reflects a worse biomarker AD profile. However, we observed no such associations with more traditional connectivity measures indicating that functional connectivity gradients are more sensitive to these early alterations.

We additionally detected differences between carriers and noncarriers of the ApoE ε4 allele. Carriers exhibited alterations in the medial parietal gradients similar to those associated with higher t-tau and p-tau as well as lower Aß/p-tau CSF levels. Earlier studies reported altered hippocampus-default mode network connectivity in ApoE ε4 healthy individuals [17], as well as a deleterious effect of this genotype on the connectivity of default mode network components [48]. Since ApoE status is tightly associated with amyloid and tau pathology [49], it is possible that the gradient changes we observed in ε4 carriers and participants with higher tau/p-tau levels and lower Aß/p-tau ratio are interlinked. Accordingly, ApoE ε4 carriers had on average higher levels of CSF total tau and lower levels of CSF Aß in the current study. However, the correlations with CSF markers remained significant after additionally correcting for ApoE status, suggesting that the associations with biomarker levels and ApoE ε4 status were at least partially independent.

We did not detect associations between cognitive performance and resting-state gradient features in the examined sample. In contrast, the identified gradients during the task-based fMRI encoding task were associated with immediate memory scores. Although functional connectivity during a goal-directed task is similar to and based on resting-state connectivity [22], task-specific connectivity modulations do occur that might reflect better the cognitive capabilities of an individual [50]. Based on these reports, it is possible that task-based gradient features capture different information from that of resting-state gradients. Indeed, task-based gradients were not associated with either CSF or genetic markers of AD in the current study indicating some level of dissociation regarding the links between resting and task-based gradients with different measures. Since several previous studies also reported different associations of task-related precuneus activity to cognitive measures and AD biomarkers, the task-based gradients might be associated with precuneal hyperactivity reported in the early stages of AD. For example, a recent study employed a novelty-related fMRI paradigm to show that precuneus activity changed in an inverted U-shape pattern across the AD spectrum in association with memory, whereas it did not correlate with CSF biomarkers [51]. Hyperactivity in the precuneus and midline regions might be associated with early tau deposition in the medial temporal lobe [52] that is not yet evident in CSF biomarker levels at this early stage, accounting for the lack of correlation with CSF biomarkers. Regarding ApoE ε4 status, studies report heterogeneous results on whether precuneus activity or connectivity differs during memory tasks. An earlier analysis of the PREVENT-AD sample reported few differences in task-related fMRI activation during memory tasks in ApoE ε4 carriers, suggesting that these participants retain memory-related functional systems at this age [29]. The observed differences in resting state gradients suggest a compensatory mechanism during task performance that might account for the dissociation of resting state and task-based gradients in connection with ApoE ε4 carriership. Alternatively, the association of task-based gradients with memory scores might reflect differences in brain development and aging that contribute to cognitive performance rather than AD-related pathology [53, 54].

## Limitations

Our study has some limitations. First, longitudinal cognitive data would be needed to investigate the value of functional gradients for the future development of AD. We will investigate this when information regarding conversion to dementia becomes available in the PREVENT-AD cohort. Furthermore, to assess whether functional gradient alterations precede or accompany amyloid or tau deposition, it would be useful to reproduce our findings in a cohort with amyloid and tau PET data. An additional issue is that, using cross-sectional data, the specificity of functional gradient alterations for disease-related processes is difficult to determine and the alterations might be related to the risk factors independently from disease mechanisms. Next, although the dominant gradients were reproducible across cohorts regardless of their fMRI parameters, because of the nonlinear nature of the decomposition, gradient changes are difficult to link to their alterations in specific connections, which is a recognized problem in the field and the reason why we did not perform analyses between the risk factors associated with AD and the connections from the medial parietal cortex to other regions [6]. Finally, although the individual reproducibility of the gradients can vary across techniques and analysis approaches, here we confirmed that individual gradients were biologically plausible by comparing them to the group average gradients and making sure that the trend surface model explained most of the variance in the individual gradients [55]. Although it is possible that the gradients differing from the group average gradient represent disease-related alterations, in the current analyses these outliers showed spatial patterns that can be considered artifactual, as they were due to a small, random population of outlier voxels, which are unlikely to follow any known disease mechanisms. Further dedicated research is needed to assess the heterogeneity of individual gradients when they do not conform to the group average gradient in terms of main direction.

## Conclusions

In summary, our results show that functional gradients of the medial parietal cortex show early alterations associated with worse CSF AD biomarkers, genetic risk factors, and subtle memory alterations, in contrast to other connectivity measures. These findings suggest that these gradients could potentially be used as a more robust and sensitive imaging marker to detect early AD signs in asymptomatic at-risk individuals before clinical disease onset.

## Supplementary Information

**Additional file 1.** Secondary analyses to demonstrate the specificity of gradient alterations. **Table S1.** Secondary analysis results. **Figure S1.** Examples of outlier functional gradients excluded from further analysis.

## Data Availability

The datasets supporting the conclusions of this article are available in the following repositories. Full imaging data from the Human Connectome Project is available at db.humanconnectome.org. Imaging and demographic data from the PREVENT-AD cohort is openly available at https://openpreventad.loris.ca, while cognitive and CSF measures are available for qualified researchers after registration at https://registeredpreventad.loris.ca.

## Abbreviations

AD: Alzheimer’s disease
CSF: Cerebrospinal fluid
fMRI: Functional MRI
Aß: Amyloid- ß_1-42_
p-tau_181_: Phosphorylated tau_181_
t-tau: Total tau
ApoE: Apolipoprotein E
RBANS: Repeatable Battery for the Assessment of Neuropsychological Status
PREVENT-AD: Presymptomatic Evaluation of Experimental or Novel Treatments for Alzheimer’s Disease
HCP: Human Connectome Project
EPI: Echo-planar imaging
MNI152: Montreal Neurological Institute 152 average standard template
FSL: FMRIB Software Library
ROI: Region of interest
TSM: Trend surface model
FDR: False discovery rate
SVD: Singular value decomposition
