# Supplementary material for: Functional gradients of the medial parietal cortex in a healthy cohort with family history of sporadic Alzheimer’s disease
Source: Alzheimers Res Ther. 2023 Apr 19;15:82. doi: 10.1186/s13195-023-01228-3 (PMC10114342; doi:10.1186/s13195-023-01228-3)
Supplement: Supplementary file 1 — Additional file 1. Secondary analyses to demonstrate the specificity of gradient alterations. Table S1. Secondary analysis results. Figure S1. Examples of outlier functional gradients excluded from further analysis. [file 13195_2023_1228_MOESM1_ESM.docx]

# **Functional gradients of the medial parietal cortex in a healthy cohort with family history of sporadic Alzheimer’s disease**

Dániel Veréb^1^, Mite Mijalkov^1^, Yu-Wei Chang^2^, Anna Canal-Garcia^1^, Emiliano Gomez-Ruis^2^, Anne Maass^3^, Sylvia Villeneuve^4,5^, Giovanni Volpe^2^, Joana B. Pereira^1^

^1^ Department of Neurobiology, Care Sciences and Society, Division of Clinical Geriatrics, Karolinska Institutet, Stockholm, Sweden

^2^ Department of Physics, Goteborg University, Goteborg, Sweden

^3^ German Center for Neurodegenerative Diseases (DZNE), Magdeburg, 39120 Magdeburg, Germany

^4^ Douglas Mental Health University Institute, McGill University, Montreal, QC, Canada.

^5^ McConnell Brain Imaging Center, Montreal Neurological Institute, McGill University, Montreal, QC, Canada

# **Supplementary material**

## **Secondary analyses to demonstrate the specificity of gradient alterations**

To assess whether traditional functional connectivity metrics can detect the same subtle changes as the functional gradients, we performed several additional analyses using the resting state fMRI scans of the PREVENT-AD cohort, namely an intrinsic functional connectivity analysis of the default-mode network (DMN), a seed-based whole-brain functional connectivity analysis based on the average medial parietal cortex ROI time series, and a seed-based whole-brain functional connectivity analysis based on the average time series of medial parietal cortex subregions. Intrinsic functional connectivity within the default mode network was calculated as the Pearson’s correlation between node time series using regions from a recent updated neuroanatomical model of the DMN (Alves *et al.*, 2019) in addition to the medial parietal cortex ROI defined in this study. Analyzed nodes included the bilateral angular gyri, superior temporal gyri, ventromedial prefrontal cortex, thalami and hippocampi, apart from the medial parietal cortex. Additionally, whole brain functional connectivity was calculated with time series extracted using the 200-region resolution Craddock-parcellation (Craddock *et al.*, 2012), using either the whole medial parietal cortex ROI or three subregions derived from the average main gradient as seeds. The latter were defined by dividing the dominant group average normalized gradient into three parts (using the thresholds <0.3, 0.3<0.7, 0.7<; gradient values in the normalized gradient range from 0 to 1). Furthermore, to determine if individual differences in subregion borders are sufficient to detect associations with markers instead of more nuanced gradient models, we calculated the relative size of subregion clusters in the medial parietal cortex by dividing individual gradients into three clusters (via the same thresholds as mentioned previously) and carried out the correlation analyses. For all the analyses, group differences were determined via independent samples Student’s t-tests and correlation analyses were carried out using the Spearman’s rank correlation coefficient. None of these analyses revealed significant associations with ApoE status, CSF markers or RBANS memory scores after multiple comparison corrections using false discovery rate (FDR). The range of statistic values and p-values are reported in **Table S1**.

**Table S1. Secondary analysis results.**

|  | ApoE ε4 carrier vs. noncarrier  (T-statistic/ p-value) | t-tau correlation (R-value/  p-value) | p-tau correlation (R-value/  p-value) | Aß42 correlation (R-value/  p-value) | Aß/p-tau ratio correlation  (R-value/  p-value) | RBANS immediate memory correlation  (R-value/  p-value) | RBANS delayed memory correlation  (R-value/  p-value) |
| --- | --- | --- | --- | --- | --- | --- | --- |
| Intrinsic DMN FC | -2.72–1.22/  0.0069-0.98 | -0.19-0.23/  0.027-0.99 | -0.15-0.20/  0.052-0.97 | -0.18-0.19/  0.077-1.00 | -0.26-0.12/  0.014-0.99 | -0.08-0.12/  0.06-0.99 | -0.07-0.10/  0.10-0.96 |
| MPC ROI – whole brain FC | -2.44-2.75/  0.0063-0.99 | -0.20-0.19/0.06-1.00 | -0.16-0.24/0.02-1.00 | -0.12-0.24/0.02-0.98 | -0.19-0.18/  0.07-1.00 | -0.13-0.15/  0.015-1.00 | -0.08-0.19/  0.003-1.00 |
| MPC subregions – whole brain FC | -3.12-3.13/  0.002-1.00 | -0.23-0.22/  0.03-1.00 | -0.21-0.26/  0.01-1.00 | -0.17-0.31/  0.003-1.00 | -0.26-0.25/  0.01-1.00 | -0.14-0.21/  0.0005-1.00 | -0.09-0.18/  0.004-1.00 |
| Relative size of MPC subregion clusters derived from individual gradients | - Cluster 1: -1.16/0.25 - Cluster 2: -1.04/0.30 - Cluster 3: 1.79/0.07 | - Cluster 1: 0.11/0.31 - Cluster 2: 0.18/0.08 - Cluster 3: -0.21/0.05 | - Cluster 1: 0.14/0.20 - Cluster 2: 0.16/0.13 - Cluster 3: -0.20/0.06 | - Cluster 1: 0.01/0.94 - Cluster 2: 0.09/0.40 - Cluster 3: -0.08/0.47 | - Cluster 1: -0.12/0,26 - Cluster 2: -0.21/0.05 - Cluster 3: 0.23/0.03 | - Cluster 1: 0.01/0.91 - Cluster 2: -0.03/0.67 - Cluster 3: 0.01/0.93 | - Cluster 1: -0.08/0.20 - Cluster 2: 0.03/0.62 - Cluster 3: 0.04/0.52 |

Abbreviations: MPC - medial parietal cortex, RBANS – Repeatable Battery for the Assessment of Neuropsychological Status, FC – functional connectivity, DMN – default mode network. All reported p-values are uncorrected for multiple comparisons. For the connectivity analyses, statistical metrics and p-values are reported as a range (minimum-maximum), referring to the range across multiple targets from a single seed ROI (in the case of MPC ROI- whole brain FC) or across pairwise differences in ROI-ROI connectivity (in the case of intrinsic DMN correlation and MPC subregions – whole brain FC).

## **
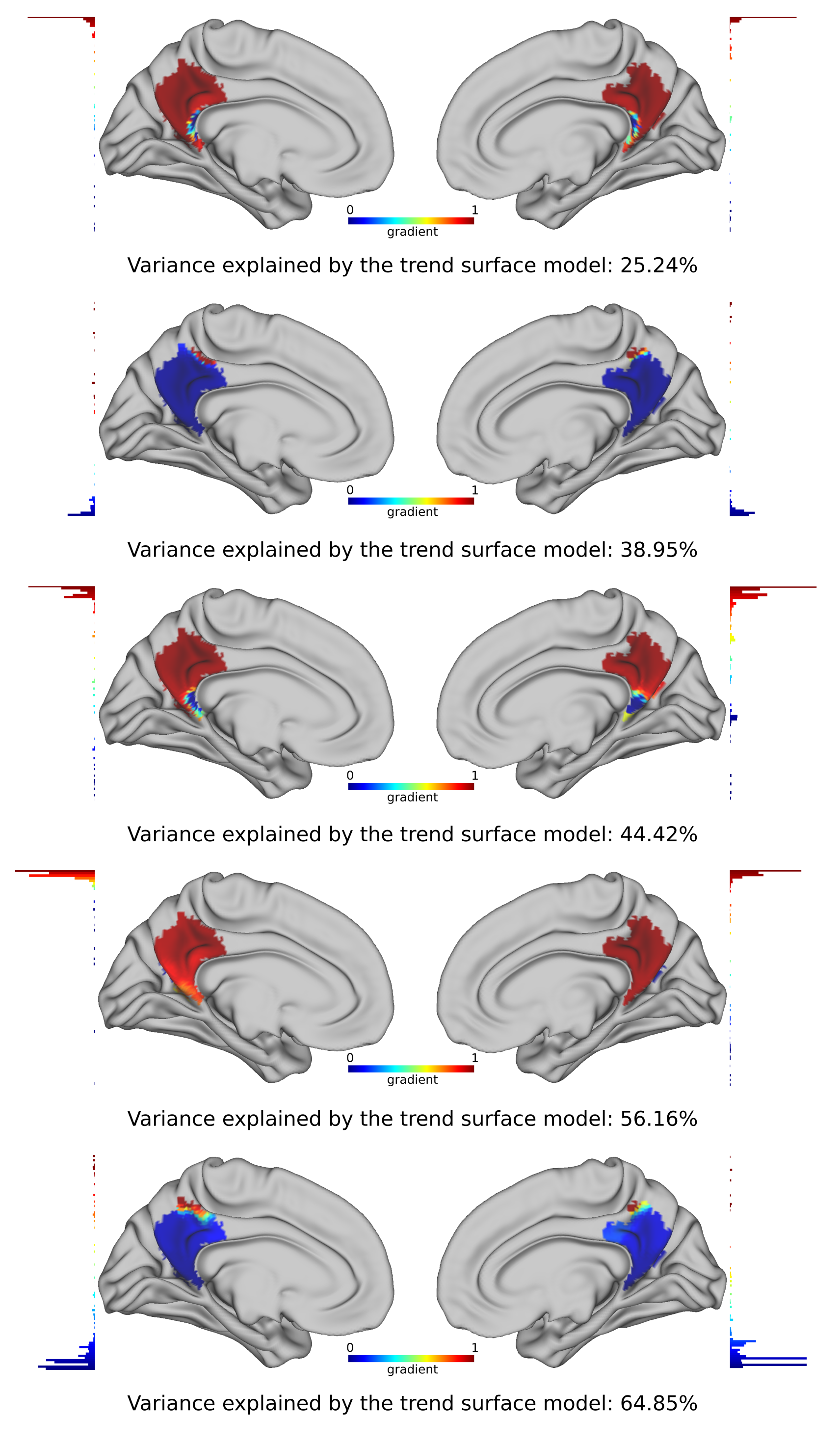
**

**Figure S1. Examples of outlier functional gradients excluded from further analysis.** Outlier functional gradients of representative participants were projected onto the HCP 32k vertex resolution surface template. These gradients, as shown by the adjacent histograms, were dominated by a small number of voxels, which were markedly different from all other voxels in the ROI, resulting in a disbalanced gradient with a steep demarcation between the outlier voxels and other voxels, while also not exhibiting clear directionality, likely representing artifactual changes rather than true connectivity distributions. The color bars denote normalized gradient values.

## **References**

Alves, P. N. *et al.* (2019) ‘An improved neuroanatomical model of the default-mode network reconciles previous neuroimaging and neuropathological findings.’, *Communications biology*, 2, p. 370. doi: 10.1038/s42003-019-0611-3.

Craddock, R. C. *et al.* (2012) ‘A whole brain fMRI atlas generated via spatially constrained spectral clustering.’, *Human brain mapping*, 33(8), pp. 1914–1928. doi: 10.1002/hbm.21333.
